# Resistance of *Pseudomonas aeruginosa* and *Staphylococcus aureus* to the airway epithelium oxidative response assessed by a cell-free *in vitro* assay

**DOI:** 10.1371/journal.pone.0306259

**Published:** 2024-08-14

**Authors:** Maïwenn Petithomme-Nanrocki, Nathan Nicolau-Guillaumet, Nicolas Borie, Arnaud Haudrechy, Jean-Hugues Renault, Sophie Moussalih, Anaëlle Muggeo, Thomas Guillard

**Affiliations:** 1 INSERM, P3Cell, U 1250, Université de Reims Champagne-Ardenne, Reims, France; 2 INSERM, CHU de Reims, Laboratoire de Bactériologie-Virologie-Hygiène hospitalière-Parasitologie-Mycologie, P3Cell, U 1250, Université de Reims Champagne-Ardenne, Reims, France; 3 CNRS, ICMR, UMR 7312, Université de Reims Champagne-Ardenne, Reims, France; Amity University Rajasthan, INDIA

## Abstract

The antibacterial oxidative response, which relies on the production of hydrogen peroxide (H_2_O_2_) and hypothiocyanite (OSCN^-^), is a major line of defense protecting the human airway epithelium (HAE) from lesions when infected. The *in vitro* studies of the oxidative responses are performed mainly by one-shot H_2_O_2_ exposure that does not recapitulate the complex H_2_O_2_/LPO/SCN^-^ system releasing the reactive oxygen species in airway secretions. A cell-free *in vitro* assay mimicking this system has been described but was not fully characterized. Here, we comprehensively characterized the hourly H_2_O_2_/OSCN^-^ concentrations produced within this *in vitro* assay and assessed the resistance of *Pseudomonas aeruginosa* and *Staphylococcus aureus* clinical strains to the HAE oxidative response. We found that H_2_O_2_/OSCN^-^ were steadily produced from 7h and up to 25h, but OSCN^-^ was detoxified in 15 minutes by bacteria upon exposure. Preliminary tests on PA14 showed survival rates at 1-hour post-exposure (hpe) to H_2_O_2_ of roughly 50% for 10^5^ and 10^7^ colony-forming unit (CFU)/mL inocula, while 10^2^ and 10^4^ CFU/mL inocula were cleared after one hpe. Thirteen clinical strains were then exposed, highlighting that conversely to *P*. *aeruginosa*, *S*. *aureus* showed resistance to oxidative stress independently of its antibiotic resistance phenotype. Our results demonstrated how this *in vitro* assay can be helpful in assessing whether pathogens can resist the antibacterial oxidative HAE response. We anticipate these findings as a starting point for more sophisticated *in vitro* models that could serve as high-throughput screening for molecules targeting the bacterial antioxidant response.

## Introduction

The lung is a complex organ composed of conducting airways and gas exchange zones. The conducting airways branch from the trachea to terminal bronchioles that end up in the alveoli. The lining epithelium provides a physical barrier between the external environment and the underlying parenchyma in the airways. The airway epithelium ensures protection of the lung against inhaled particles, toxins, and pathogens through the mucociliary clearance and secretion of molecules with antibacterial, antioxidant, and antiprotease activity that act in an orchestrated way to protect the epithelium from lesion factors. The antibacterial oxidative response, which is a major innate defense system, relies on the production of hydrogen peroxide (H_2_O_2_) and hypothiocyanite (OSCN^-^) by DUOX enzymes (DUOX 1 and 2) and lactoperoxidase (LPO) [1, 2]. DUOX enzymes continuously generate H_2_O_2_ at the apex of the airway epithelial cells (AEC) toward the airways’ lumen. This damaging product for AEC [3, 4] is thereby quickly used by LPO to oxidize thiocyanate (SCN) into OSCN^-^ [5]. SCN^-^, provided by feeding, is present in numerous body fluids [6]. Therefore, the antibacterial oxidative response stems from the H_2_O_2_/LPO/SCN^-^ system releasing these two reactive oxygen species (ROS) in airway secretions.

**Fig 1:**
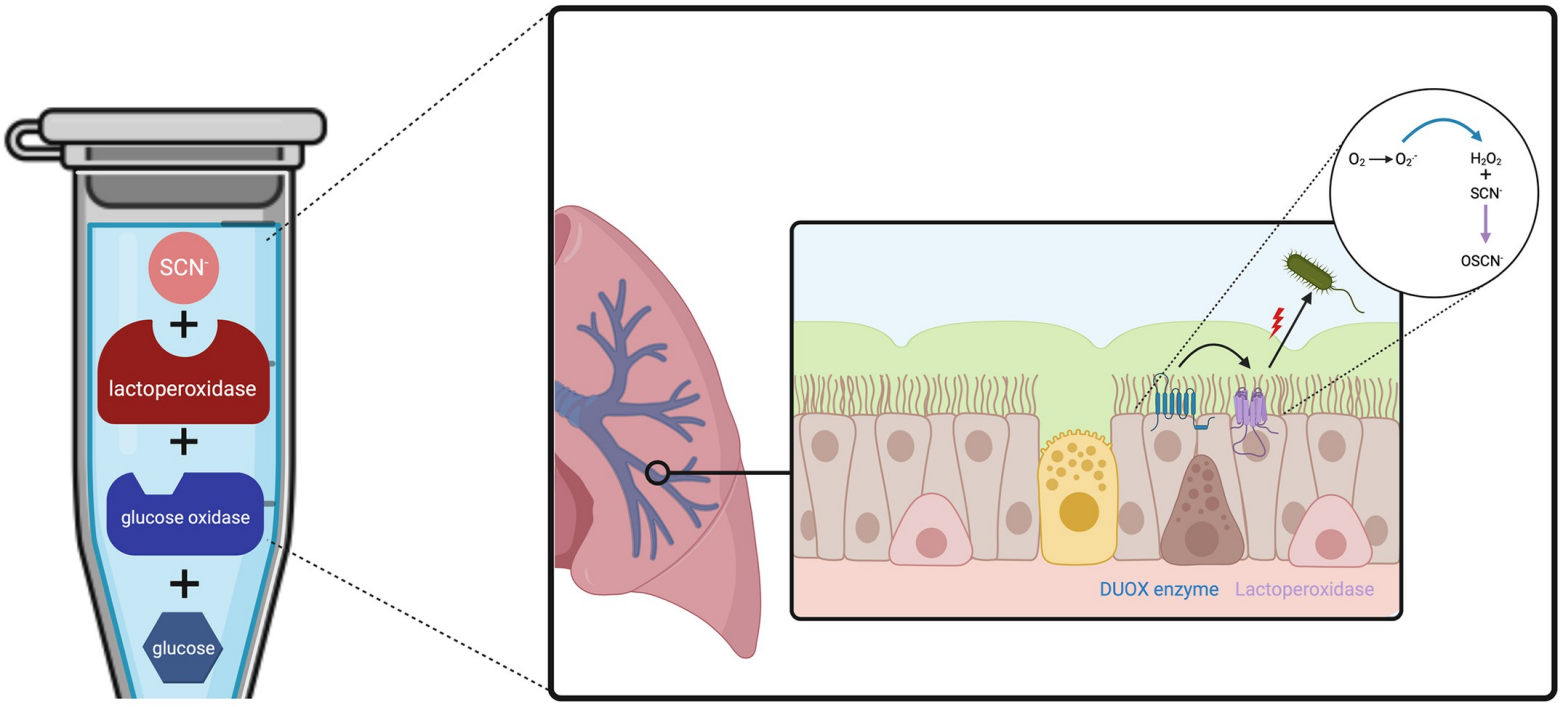
Cell-free *in vitro* H_2_O_2_/LPO/SCN^-^ assay mimicking the antimicrobial oxidative response of the Human Airway Epithelium (HAE). This diagram summarizes how the *in vitro* test mimics the oxidative response of the HAE.

Oxidative stress, and more precisely H_2_O_2_ solely and H_2_O_2_/LPO/SCN^-^ system, have been studied for their antibacterial effect [7, 8]. Indeed, the survival of several pathogens upon exposure to various durations and intensities of ROS has been investigated. Notably, these experiments mostly consisted of one H_2_O_2_ shot, limiting them to the abrupt addition of high amounts of H_2_O_2_ from millimoles to moles [7, 9–13]. Such assays do not seem to mimic the *in vivo* context. H_2_O_2_ *in vitro* half-time life is around 10^−3^ seconds, prevailing a reliable assessment of the precise concentration in the airways [4]. However, the condensation of exhaled air with liquid nitrogen and the air-liquid interface epithelium models provided trends of H_2_O_2_ concentrations, ranging from micromoles to millimoles [14, 15]. With H_2_O_2_ added all at once at a specific concentration, tested pathogens are exposed to a likely higher starting concentration (from 0.19 to 7.5 mM [7, 13, 16]) than in the airways and for a shorter period. To our knowledge, only one *in vitro* assay has been set up to better recapitulate the H_2_O_2_/LPO/SCN^-^ *in vivo* system, allowing the continuous and slow production of H_2_O_2_ and OSCN^-^ [17]. This cell-free *in vitro* assay described by Patel *et al*. was successfully used on influenza A and B viruses and showed that the LPO-based system is a key player in the antiviral defense within airways. Although the experimental set-up clearly described the concentrations of each reactive inducing the production of H_2_O_2_ and OSCN^-^, no data were shown for the hourly H_2_O_2_ and OSCN^-^ concentrations obtained. Such a lack of data precludes a reliable use of the cell-free *in vitro* system to mimic the antibacterial oxidative response of the HAE. For instance, any scientist willing to use such an assay would have to address two relevant questions: (i) when should we start the oxidative exposure of pathogens after initiating the enzymatic production of H_2_O_2_/OSCN^-^ and (ii) how long does the slow but maintained *in vitro* H_2_O_2_/OSCN^-^ kinetic last? For these reasons, we aimed to comprehensively characterize the cell-free *in vitro* H_2_O_2_/LPO/SCN^-^ system by providing a detailed protocol, the hourly H_2_O_2_ and OSCN^-^ concentration produced, and experimental methods to determine those parameters. First, we set up an H_2_O_2_ producing model and the experimental H_2_O_2_/LPO/SCN^-^ system using the laboratory strain *Pseudomonas aeruginosa* PA14. Second, as *P*. *aeruginosa* and *Staphylococcus aureus* are leading causes of nosocomial pneumonia [18], we used our cell-free system (Fig 1) to assess the resistance of clinical strains.

## Material and methods

### Bacterial strains

The laboratory strain PA14 was used to set up our protocol. After optimization and determination of the H_2_O_2_/OSCN^-^ concentrations produced, we exposed seven various strains of *P*. *aeruginosa* and *S*. *aureus* upon H_2_O_2_/OSCN^-^. For *P*. *aeruginosa*: one reference ATCC 27853 strain and six clinical isolates. For *S*. *aureus*: two reference strains (Methicillin-susceptible *S*. *aureus* [MSSA, ATCC 20213] and Methicillin-resistant *S*. *aureus* [MRSA, NCTC 12493] and five clinical isolates (3 MSSA and 2 MRSA). The microbiological characteristics of clinical strains selected for the exposure are listed in Table 1. Bacteria were incubated overnight at 37°C in Lysogeny broth (LB) under permanent shaking at 250 rpm. Bacterial solutions at 10^7^ colony-forming unit (CFU)/mL, 10^5^ CFU/mL, 10^4^ CFU/mL, and 10^2^ CFU/mL were obtained from sequential dilutions of a 10^9^ CFU/mL suspension in Dulbecco’s Phosphate Buffered Saline (DPBS; Sigma Aldrich, France), determined with an absorbance measurement of 1.00 at an optical density of 600 nm wavelength (OD_600_).

**Table 1:** Microbiological characteristics of clinical strains used in the experiment.

Species	Sample	Ward	Resistance phenotype	Reference
*S*. *aureus*	NA		MS	ATCC 20213
			MR	NCTC 12493
	Bronchoalveolar lavage	ICU	MS	RSR 20220617
	Sputum culture	CF	MS, Ery^R^, Cl^R^	RSR 20220616
		CF	MS, Ery^R^, Cl^R^, FQ^R^, Tob^R^, Tet^R^, Fus^R^, Rif^R^	RSR 20220618
	Aspiration	ICU	MR, FQ^R^	RSR 20220620
	Sputum culture	CF	MR, Gen^R^, Tob^R^	RSR 20220619
*P*. *aeruginosa*	NA		Susceptible	ATCC 27853
	Sputum culture	CF	Susceptible	RSR 20220609
	Bronchoalveolar lavage	ICU	Imi^R^	RSR 20220610
		ICU	ESBL	RSR 20220611
		ICU	Carbapenemase VIM	RSR 20220612
	Sputum culture	CF	Pan-drug resistant	RSR 20220911

CF: cystic fibrosis follow-up, Cl^R^: resistant to clindamycin, Ery^R^: resistant to erythromycin, ESBL: extended-spectrum beta-lactamase, Fus^R^: resistant to fusidic acid, FQ^R^: resistant to levofloxacin, Gen^R^: resistant to gentamicin, ICU: intensive care unit, Imi^R^: resistant to imipenem solely, MS: Methicillin-susceptible, MR: Methicillin-resistant, NA: not applicable, Rif^R^: resistant to rifampicin, Tet^R^: resistant to tetracycline, Tob^R^: resistant to tobramycin, VIM: Verona IMipenemase.

### High-resolution mass spectrometry

Direct infusion MS analysis was performed on a Waters Acquity UPLC system coupled with a Waters SYNAPT G2-Si High-Resolution Mass Spectrometry equipped with electrospray ionization (ESI) source (Waters Corp., Manchester, UK). Mass detection was conducted in the negative ion mode, with the source temperature at 100°C, and the capillary voltage and cone voltage were set at 2 kV and 40 V, respectively. The desolvation gas was optimized to 650 L/h, the cone gas flow was 50 L/h, and the scan range was from 50 to 1600 m/z. Mass was corrected during acquisition using an external reference (Lock-Spray) consisting of a 1 ng/μL solution of leucine enkephalin at a flow rate of 5 μL/min to ensure accuracy and reproducibility during the MS analysis. All data collected were acquired using MassLynx^™^ (V4.1) software in continuum mode.

### Cell-free assays

First, we set up a H_2_O_2_ production assay (S1A Fig). The model producing continuous H_2_O_2_ was composed of glucose oxidase (Sigma Aldrich, France) and D-glucose (VWR International, France) at concentrations of 0.01 U/mL and 5 mmol/L, respectively. Equal volumes of solutions at 0.02 U/mL and 10 mmol/L, respectively, were then added to obtain the concentrations initially mentioned. They were assembled in a sterile opaque Eppendorf tube at 4°C. Second, based on the continuous production of H_2_O_2_, we set up an OSCN^-^ production assay (S1B Fig). The model producing continuous OSCN^-^ has four components: LPO (Sigma Aldrich, France), glucose oxidase, D-glucose and SCN^-^/K^+^ (Sigma Aldrich, France) at the respective concentrations of 6.5 μg/mL, 0.01 U/mL, 5 mmol/L and 400 μmol/L. To obtain 4 mL of OSCN^-^-producing solution, 200 μL of glucose oxidase 0.2 UI/mL, 200 μL of D-glucose 100 mM, 200 μL of KSCN 8 mM and 130 μL of LPO 200 μg/mL were added to 3,270 μL of PBS. As for H_2_O_2_, all the reactives were assembled in a sterile opaque Eppendorf tube at room temperature. The 2-nitro-5-thiobenzoic acid (TNB) was used to measure the OSCN^-^ produced. Therefore, the H_2_O_2_ and OSCN^-^ producing solutions were used to fill wells for bacterial exposure.

### Measurements of H_2_O_2_ concentration

The ROS-Glo^™^ H_2_O_2_ Assay from Promega was used at 1, 3, 5, 7, 17 and 23 hours post-production (hpp) to determine the H_2_O_2_ concentration obtained. It is based on the transformation of H_2_O_2_ into luciferin precursor and then luciferin as the terminal product of luciferase. The catalyzation of luciferin by luciferase emits a luminescence correlated with the amount of H_2_O_2_. Briefly, 50 μL of our D-glucose and glucose oxidase sample were incubated for 1 hour with 20 μL of H_2_O_2_ substrate solution. Then, 100 μL of ROS-Glo^™^ Detection Solution was added and incubated for 20 minutes at room temperature. The Tecan Spark^™^ multimode microplate reader (Tecan, Austria) was used to measure the luminescence. The calibration curve was obtained with 30% H_2_O_2_ solution (Sigma Aldrich, France) diluted from 500 to 10 mmol/L in PBS.

### Measurement of OSCN^-^ concentrations using TNB

TNB is an acid that turns orange to colorless when binding sulfhydryl groups. The production process of TNB and, after that, its use for measurement of OSCN^-^ is represented in S2 Fig. TNB was obtained by mixing 40 mg of 5,5’-dithiobis-(2-nitrobenzoic acid) (DTNB, Sigma Aldrich) with 20 mg of sodium borohydride (NaBH_4_, Sigma Aldrich) in 100 mL of Tri-HCl buffer 0.5 mol/L pH 7. As expected, it was found to have the molecular formula C_7_H_5_NO_4_S, (HRESIMS *m/z* 197.80 [M-H]^-^, calcd for 197.99) (S3A Fig). It was then stored at 4°C in the dark for up to seven days. DTNB was obtained with the molecular formula C_14_H_6_N_2_O_8_S_2_, (HRESIMS *m*/*z* 394.96 [M-H]^-^, calcd 394.96) (S3B Fig). It was later used as a reference to assess the quality of the TNB produced. TNB is orange, and its absorbance can be detected at a wavelength of 412 nm. It has a sulfide radical that can bind to molecules containing another sulfide radical to form a new disulfide bond, losing its orange colour. The concentration can then be deduced with the Beer-Lambert law equation using the molar extinction coefficient of TNB 14,150 L.mol^-1^.cm^-1^.

OSCN^-^ was measured at 1, 3, 5, 7, 17, 23, 30 and 34 hpp. First, the production of H_2_O_2_ and subsequently OSCN^-^ was instantly stopped by adding 300 μL of catalase 700 UI/mL to 600 μL of the samples. After vortexing, 500 μL of TNB were added before a second vortexing. A 96-well transparent plate with flat bottoms (Falcon^®^, France) was filled with 200 μL of this solution, and absorbance was measured with Tecan Spark^™^ at 412 nm wavelength. A control for initial absorbance was obtained by filling 200 μL in a well from a control solution (900 μL of PBS with 500 μL of TNB). Since our 96-well transparent plates have a different diameter at the top and the bottom of each well, two concentrations have been determined using two different heights of liquid crossed by the spectrophotometer beam. Indeed, the exact height filled with 200 μL of the solution was unavailable. We calculated concentrations for two heights, a maximum and a minimum one, with the volume equation of a cylinder and the two diameters given by the manufacturer (Eq 1).


$$\frac{\Delta A}{\varepsilon \pi r_{2}^{2}}<c<\frac{\Delta A}{\varepsilon \pi r_{1}^{2}}$$

Eq 1.**The equation used to determine the OSCN^-^ concentrations based on absorbance measured.** The concentration was calculated using the Beer-Lambert law equation with the molar extinction coefficient (ε), length crossed by the beam (l) and the difference in absorption between TNB with and without the sample (ΔA).

### Measurement of OSCN^-^ concentrations upon bacteria exposure

The OSCN^-^ production was assessed while bacteria were exposed to this oxidant within the *in vitro* assay. We measured its concentration after 10 and 15 hpp upon exposure to 10^2^ and 10^7^ CFU/mL PA14 for 15 minutes, 1 hour post exposure (hpe) and 6 hpe. In a 96-well plate, each well was filled with 45 μL of bacteria and 5 μL of OSCN^-^-producing solution. For each time and each starting inoculum, 3 replicates were performed. Therefore, we pooled the 3 wells in a 1.5 mL Eppendorf and centrifugated it for 3 minutes at 13,000 rpm. The supernatant was then collected, and 75 μL of 700 UI/mL catalase was added to decompose the H_2_O_2_ produced. After vortexing, 125 μL of TNB were then added to the solution that was vortexed again. In a 96-well flat bottom transparent plate, 200 μL of the final solution were then filled in, and absorbance at 412 nm was acquired in the Tecan Spark^™^. The concentration of OSCN^-^ was deduced as previously described.

### H_2_O_2_/LPO/SCN^-^ *in vitro* assay of *P*. *aeruginosa* and *S*. *aureus* strains

In wells with 5 μL of H_2_O_2_ or OSCN^-^-producing solution, we added 45 μL of the bacterial suspension. Taking into account the dilution to the tenth, the concentrations used were D-glucose 100 mmol/L, glucose oxidase 0.2 U/mL for the H_2_O_2_-producing mix (incubated at 4°C for 18h) and D-glucose 1 mol/L, glucose oxidase 2 U/mL, LPO 2 mg/mL and KSCN 800 mmol/L for the OSCN^-^-producing mix (incubated at room temperature for 18h). A 96-well sterile plate was used for this experiment. A well was filled in with an inoculum for each time of interest (in triplicate). A well-filled in with only bacterial solution was considered as the negative control. Survival ratios were then calculated by dividing CFU from H_2_O_2_ exposed wells over CFU from the negative control. The incubation was performed sheltered from light. After exposure for the duration predefined, 10 μL of the solution was diluted in PBS for the 10^7^ and 10^5^ inocula or directly inoculated on an LB agar plate. Bacteria colonies were counted after 17h of incubation, and survival rates were calculated. Ten biological replicates were realized for each strain.

### Statistics

For survival rates within the H_2_O_2_/LPO/SCN^-^ *in vitro* assays of each strain, a two-way ANOVA with a Dunn’s multiple comparisons test was used to compare the measured values between the different inocula and time. The error bars represent a 95% confidence interval (IC95). An unpaired non-parametric Mann- Whitney U test was carried out to compare the results of two strains (at one time and one inoculum). The statistical significance was considered for *p* < 0.05. All the tests were performed using GraphPad Prism version 9.

## Results

### H_2_O_2_/OSCN^-^ are steadily produced from 7h and up to 25h within the H_2_O_2_ and H_2_O_2_/LPO/SCN^-^ *in vitro* assays

**Fig 2:**
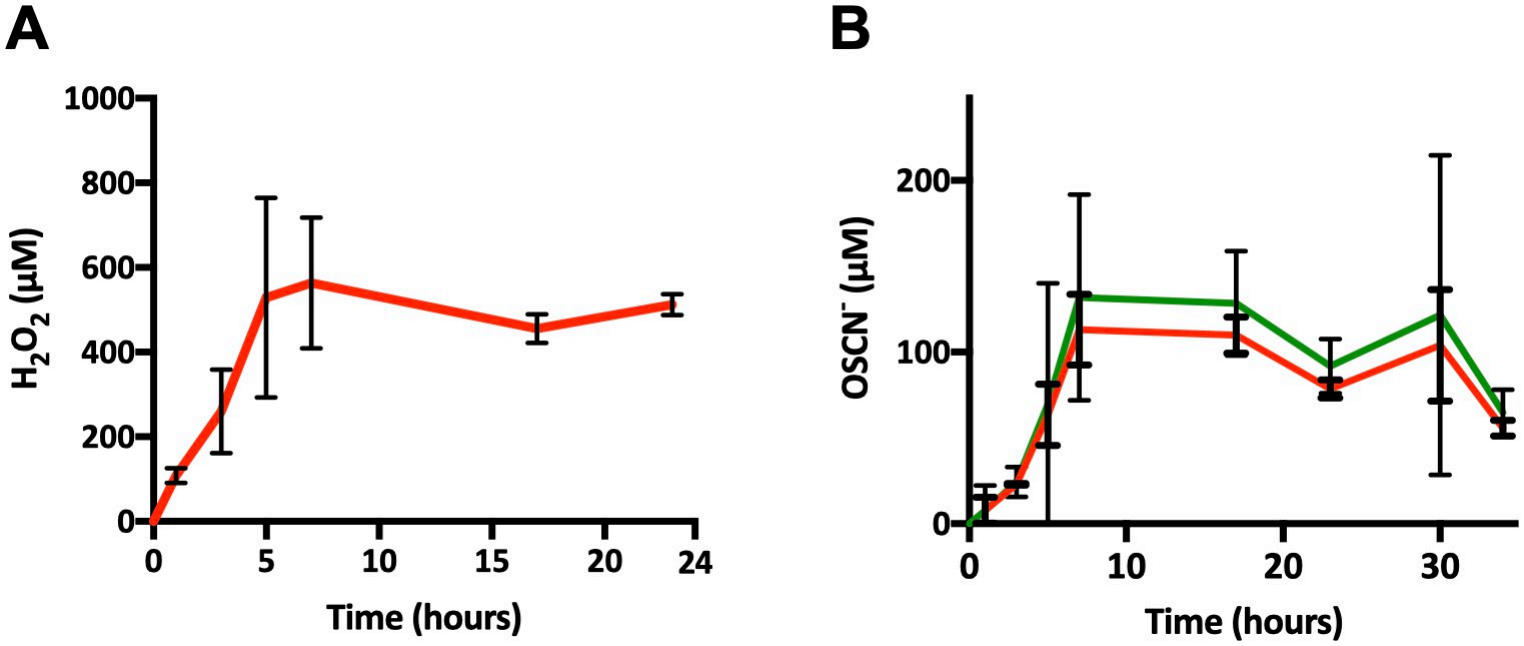
Kinetic of H_2_O_2_ and OSCN^-^ production within the *in vitro* cell-free assays. The curves of H_2_O_2_ (A) during the H_2_O_2_ production assay and OSCN^-^ (B) during the OSCN^-^ production assay, obtained after dosage with ROS-Glo^™^ H_2_O_2_ assay and TNB assay, n = 3 (concentrations calculated for the minimum (green) and maximum (red) height in the well). All relevant data and statistics description are within the supporting information and excel files.

We dosed separately and iteratively H_2_O_2_ and OSCN^-^ during their production within both assays. We showed a constant production of those two key players in the antibacterial oxidative response. We obtained an increased amount of H_2_O_2_ and OSCN^-^ in the first 7h with a steady state for around 24h (Fig 2). For H_2_O_2_, the concentration reached about 500 μM at 7h and was maintained for up to 23h (Fig 2A). The production of OSCN^-^ had the same dynamic as H_2_O_2_ and presented a constant increase up to 7h with a steady state around 150 μM (CI: 72;191) up to 30h (Fig 2B).

### OSCN^-^ is detoxified by bacteria within the H_2_O_2_/LPO/SCN^-^ *in vitro* assay

**Fig 3:**
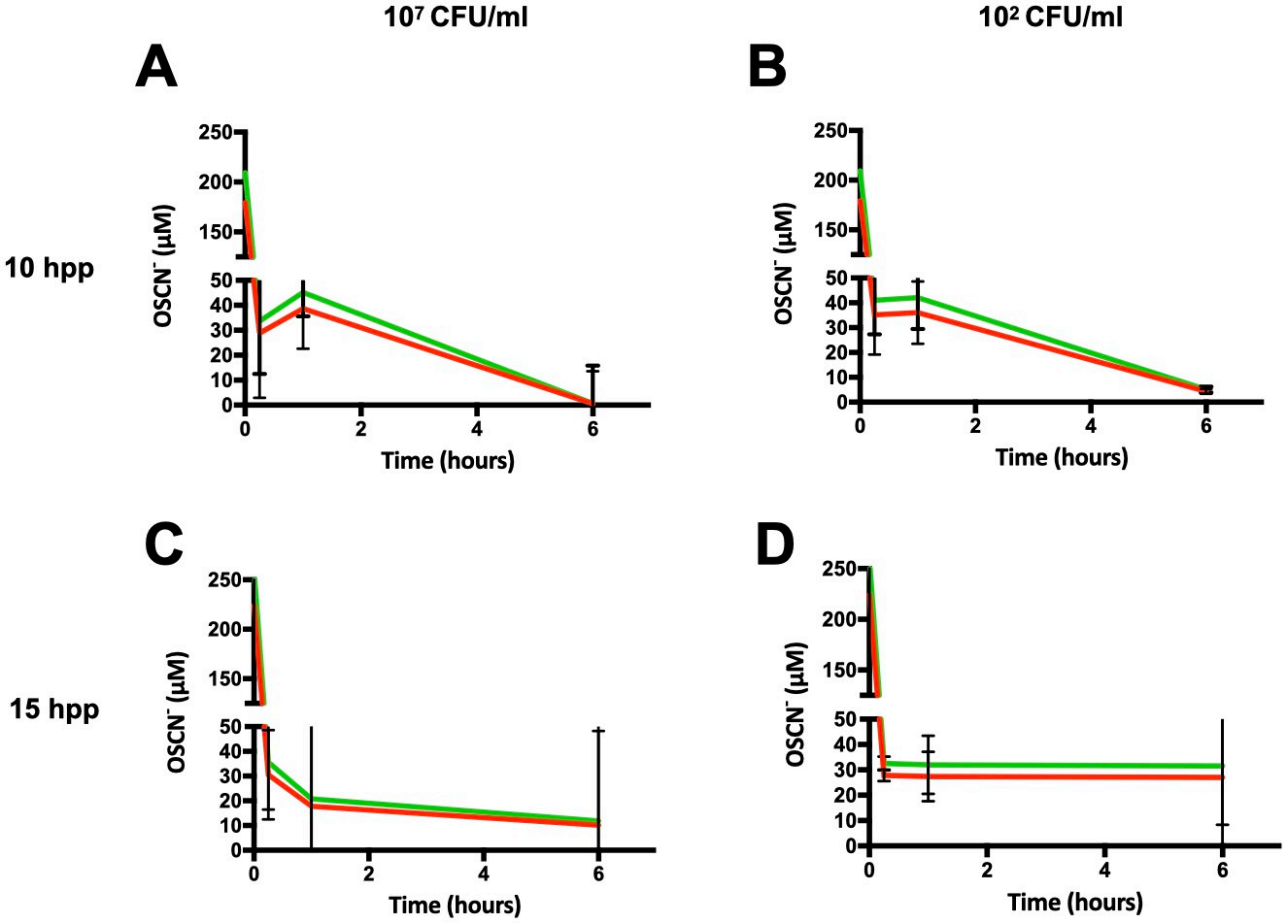
Kinetic of OSCN^-^ production within the *in vitro* cell-free assay upon bacteria exposure. Curves of OSCN^-^ obtained after dosage with TNB. PA14 inoculum of (A) 10^7^ CFU/mL or (B) 10^2^ CFU/mL after 10 hpp (n = 3). PA14 inoculum of (C) 10^7^ CFU/mL or (D) 10^2^ CFU/mL after 15 hpp (n = 3). Concentrations were calculated for the minimum (green) and maximum (red) height in the well. All relevant data and statistics description are within the supporting information and excel files.

First, TNB was synthesized from DTNB by NaBH_4_ reduction as described in the Material and Methods section. To assess whether OSCN^-^ production was steady or decreased due to detoxification by exposed bacteria during the *in vitro* OSCN^-^ production assay, we measured OSCN^-^ concentration after 10 hpp and 15 hpp to PA14 (see Material and methods section). As shown in Fig 3, for both, we found a dramatic 85% decrease of the OSCN^-^ concentration as earlier as 15 minutes of 10^7^ CFU/mL (Fig 3A and 3C) or 10^2^ CFU/mL (Fig 3B and 3D) bacterial exposure. The 35 μM remaining OSCN^-^ was then stable up to 1h of exposition. At 6h, OSCN^-^ was eventually nearly undetectable.

### Validation of the H_2_O_2_/LPO/SCN- *in vitro* assays as a bacterial inactivation test

**Fig 4:**
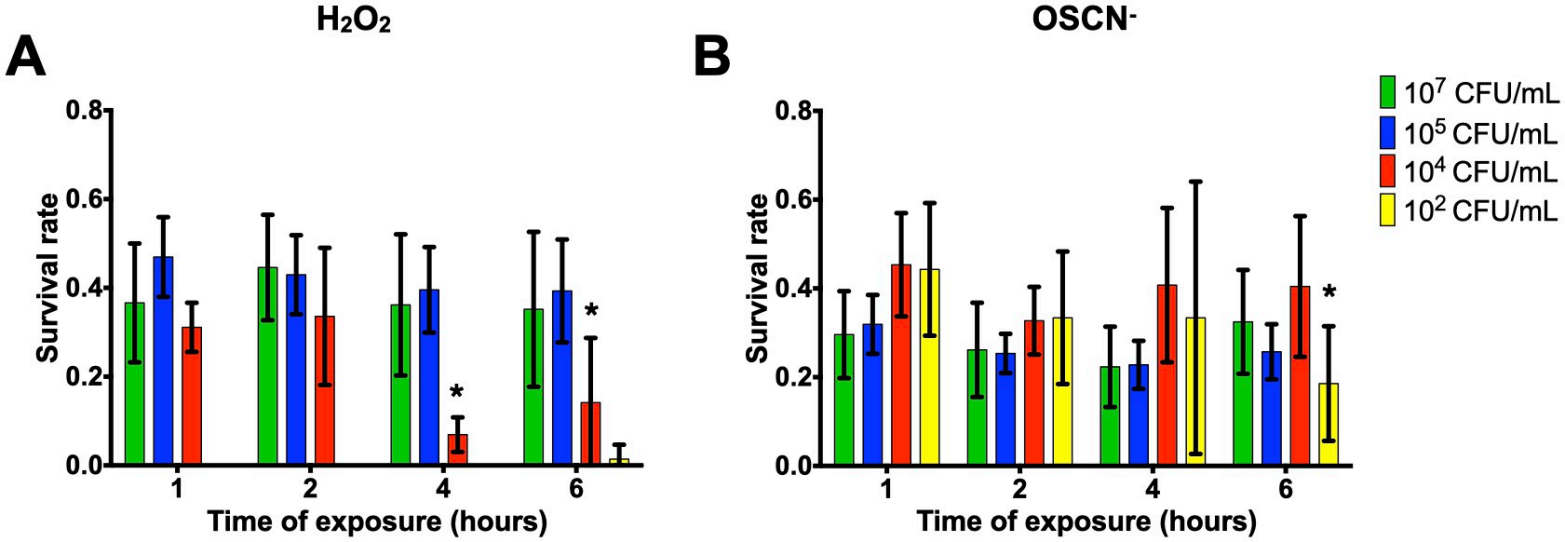
Survival rates of PA14 within the H_2_O_2_/LPO/SCN- *in vitro* assays. PA14 was incubated in the presence of **(A)** H_2_O_2_ or **(B)** OSCN^-^ for 1h, 2h and 6h. The bacterial survival was assessed by CFU counting. Mean +/- CI95, n = 10. Data were analysed using a 2-way ANOVA with an P value < 0.05 for inocula as a source of variation in the overall ANOVA. *P <0,05 using Dunnett’s multiple comparisons test. All relevant data are and statistics description within the supporting information and excel files.

Given the kinetic of H_2_O_2_/OSCN^-^ production in the two assays, we started exposing the bacteria after a minimum of 7 hpp of H_2_O_2_ or OSCN^-^ production assay. The PA14 strain was first tested to work out the feasibility of our assay. H_2_O_2_ fully inactivated the lower inoculum 10^2^ CFU/mL after 1h. For H_2_O_2_, a statistically significant decrease was found upon exposure for the 10^4^ CFU/mL inoculum (Fig 4A). The survival rate of a 10^4^ CFU/mL inoculum decreased drastically after 4h with less than 10% of bacterial survival. For higher inocula (10^5^ and 10^7^ CFU/mL), the survival rates of the bacteria constituting the infectious inoculum upon H_2_O_2_ exposure were less than 50%, but no statistically significant decrease was evidenced through the hours post-exposure. The survival rates for OSCN were stable throughout the 6 hpe for all inocula except for the 10^2^ CFU/mL, which showed a significant decrease at 6 hpe (Fig 4B). Conversely to H_2_O_2_, OSCN^-^ did not inactivate the low 10^2^ CFU/mL inoculum (Fig 4B, yellow bars). The survival rates to OSCN^-^ inactivation varied between 70 and 50% with all inocula (Fig 4B). Although PA14 seemed more capable of withstanding the action of OSCN^-^ than H_2_O_2_, no statistically significant difference was found at the different hpe or each inoculum.

### Conversely to *P*. *aeruginosa*, *S*. *aureus* seems to be resistant to oxidative stress independently of its antibiotic resistance phenotype

**Fig 5:**
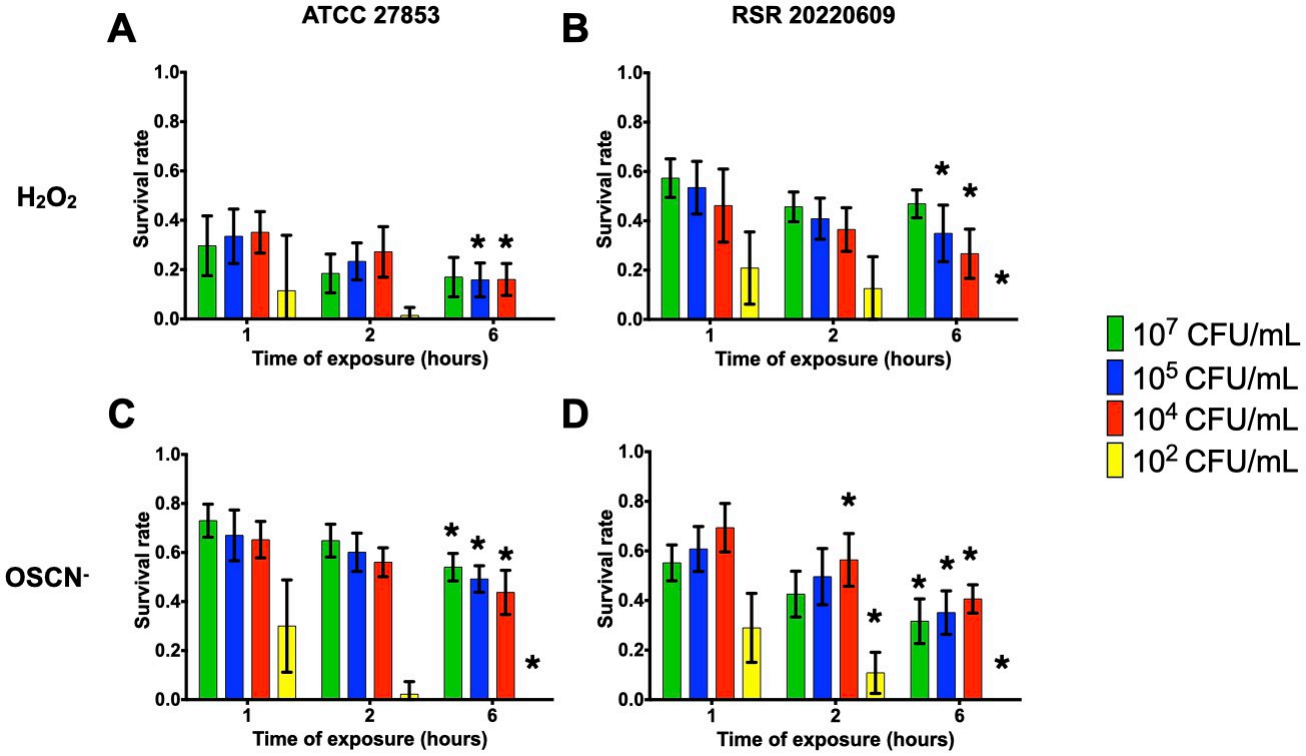
The survival rate of reference and susceptible clinical *P*. *aeruginosa* strains within the H_2_O_2_ and the H_2_O_2_/LPO/SCN^-^ *in vitro* assays. The bacterial strains were incubated in the presence of **(A and B)** H_2_O_2_ or **(C and D)** OSCN^-^ for 1h, 2h and 6h. The bacterial survival was assessed by CFU counting. Mean +/- CI95, n = 10. Data were analysed using a 2-way ANOVA with an *P* value < 0.05 for inocula as a source of variation in the overall ANOVA. *P <0,05 using Dunnett’s multiple comparisons test. All relevant data and statistics description are within the supporting information and excel files.

**Fig 6:**
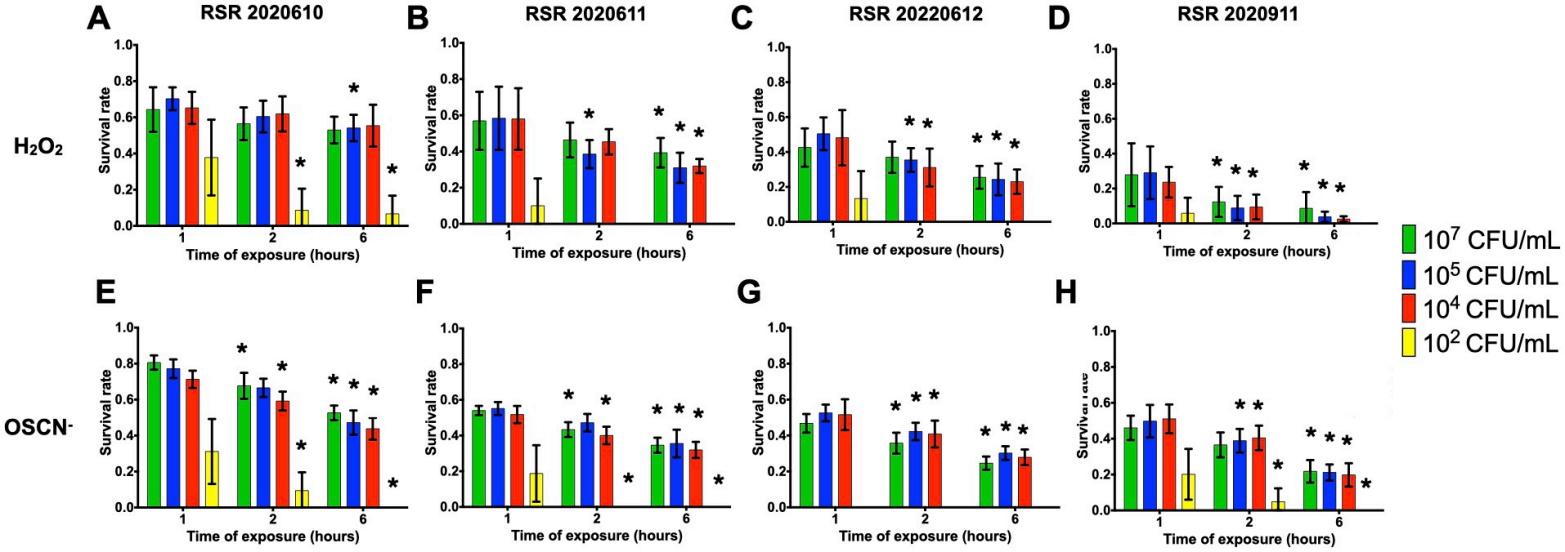
Survival rate of resistant clinical *P*. *aeruginosa* strains within the H_2_O_2_ and the H_2_O_2_/LPO/SCN^-^ *in vitro* assays. The bacterial strains were incubated in the presence of **(A to D)** H_2_O_2_ or **(E to H)** OSCN^-^ for 1h, 2h and 6h. The bacterial survival was assessed by CFU counting. Mean +/- CI95, n = 10. All relevant data and statistics description are within the supporting information and excel files.

**Fig 7:**
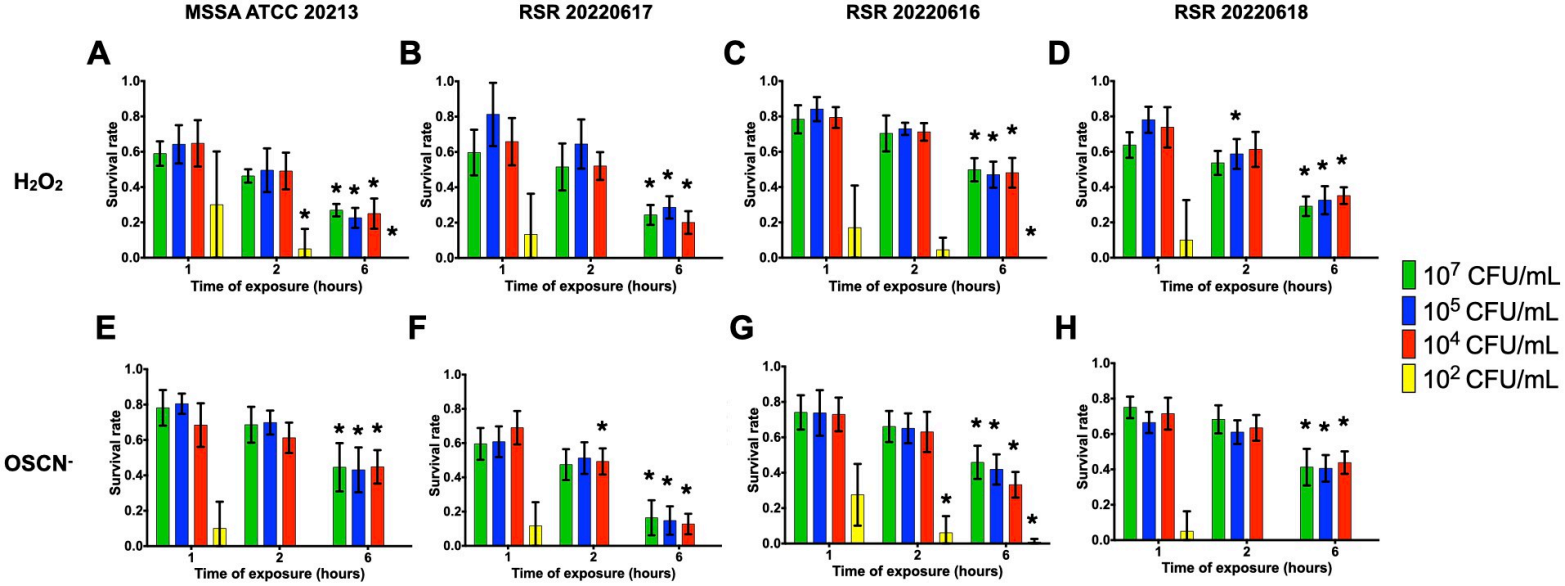
Survival rates of reference and clinical methicillin-susceptible *S*. *aureus* strains within the H_2_O_2_ and H_2_O_2_/LPO/SCN^-^ *in vitro* assays. The bacterial strains were incubated in the presence **of (A to D)** H_2_O_2_ or **(E to H)** OSCN^-^ for 1h, 2h and 6h. The bacterial survival was assessed by CFU counting. Mean +/- CI95, n = 10. MSSA: Methicillin-susceptible *S*. *aureus*. All relevant data and statistics description are within the supporting information and excel files.

**Fig 8:**
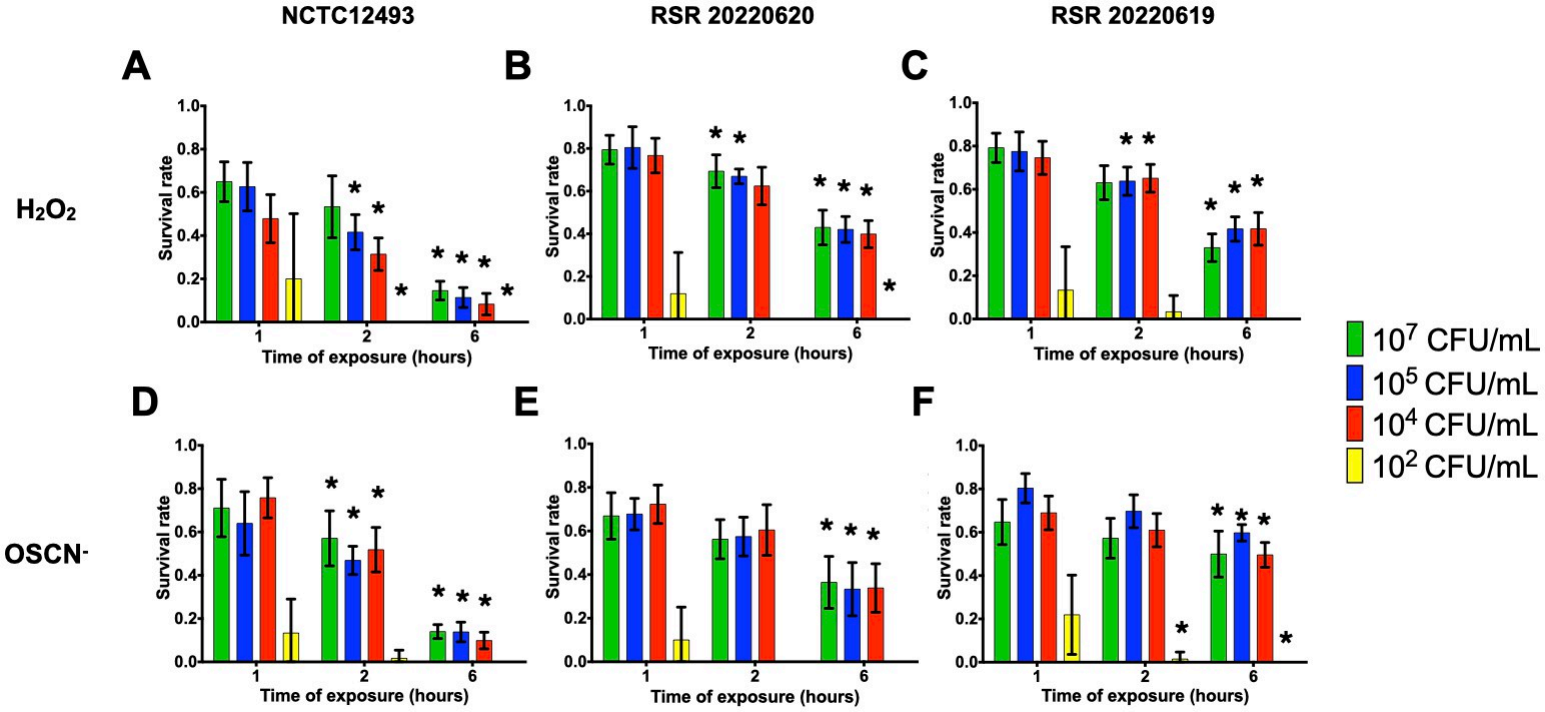
Survival rates of reference and clinical methicillin-resistant *S*. *aureus* strains within the H_2_O_2_ and H_2_O_2_/LPO/SCN^-^ *in vitro* assays. The bacterial strains were incubated in the presence of (A to C) H_2_O_2_ or (D to F) OSCN^-^ for 1h, 2h and 6h. The bacterial survival was assessed by CFU counting. Mean +/- CI95, n = 10. MRSA: Methicillin-resistant *S*. *aureus*. All relevant data and statistics description are within the supporting information and excel files.

Taking into account the results for the survival of PA14, the survival rates of *P*. *aeruginosa* and *S*. *aureus* clinical strains were assessed with inoculum ranging from 10^2^ to 10^7^ CFU/mL and CFU counting were performed at 1, 2 and 6 hpe of H_2_O_2_ and OSCN^-^. For most of the inocula tested, *S*. *aureus* or *P*. *aeruginosa* strains showed a significant decrease when comparing survival rates at 6 hpe compared to 1 hpe. (Figs 5–8). Regardless of the resistance phenotype, these results confirmed that H_2_O_2_ and OSCN^-^ significantly affected bacterial growth.

All *P*. *aeruginosa* strains showed a better survival rate when exposed to OSCN^-^ than when exposed to H_2_O_2_ (Figs 5 and 6). Regardless of the inoculum tested, all the strains showed decreased survival. The most significant difference between the H_2_O_2_ or OSCN^-^ exposure was evidenced with the ATCC 27853 strain (Fig 5A and 5D). This strain exposed to H_2_O_2_ showed a survival rate of 30% after 1h, which decreased to 20% after 6h. Upon OSCN^-^ exposure, we found a 70% survival rate, with a drop to 60% after 6h. This trend was observed for the 10^7^, 10^5^ and 10^4^ CFU/mL inocula. Interestingly, the 10^2^ CFU/mL inoculum did not survive upon any oxidative exposure, except for 1h of OSCN^-^; for the susceptible clinical strains (RSR 20220609, RSR 20220610), we observed a survival rate to H_2_O_2_ or OSCN^-^ ranging from 60 to 40% (Fig 5) whereas the three resistant strains (RSR 20220611, RSR 20220612, RSR 20220911) ranged from 40% to almost no survival (Fig 6). However, no statistically significant difference was found between the strains at 1 hpe.

For *S*. *aureus* clinical strains, no survival was observed for the 10^2^ CFU/mL inoculum (Figs 7 and 8). For the other inocula, *S*. *aureus*, either methicillin-resistant or susceptible strains, presented survival rates of roughly 70% after 1h of exposure to either H_2_O_2_ or OSCN^-^ The survival rates dropped off around 10% lower after 2 hpe. Every strain showed a decrease during the assay. At 6 hpe, we found survival rates ranging from 20% to 40%, depending on the strain. The ATCC MSSA and MRSA reference strains (Figs 7A–7E and 8A–8D) showed a trend of survival rates lower than clinical strains. No statistically significant difference was found between the strains at 10^5^ CFU/mL at 1 hpe.

Although not statistically significant, a trend was observed for *S*. *aureus* and *P*. *aeruginosa* strains depending on their antibiotic resistance phenotype. Indeed, the survival rate appeared to decrease as antibiotic resistance increased for *P*. *aeruginosa*. The more resistant the strains, the less they withstood oxidative stress.. For *S*. *aureus*, the survival rate was similar whether the phenotype of resistance was methicillino-resistant or susceptible.

## Discussion

**Fig 9:**
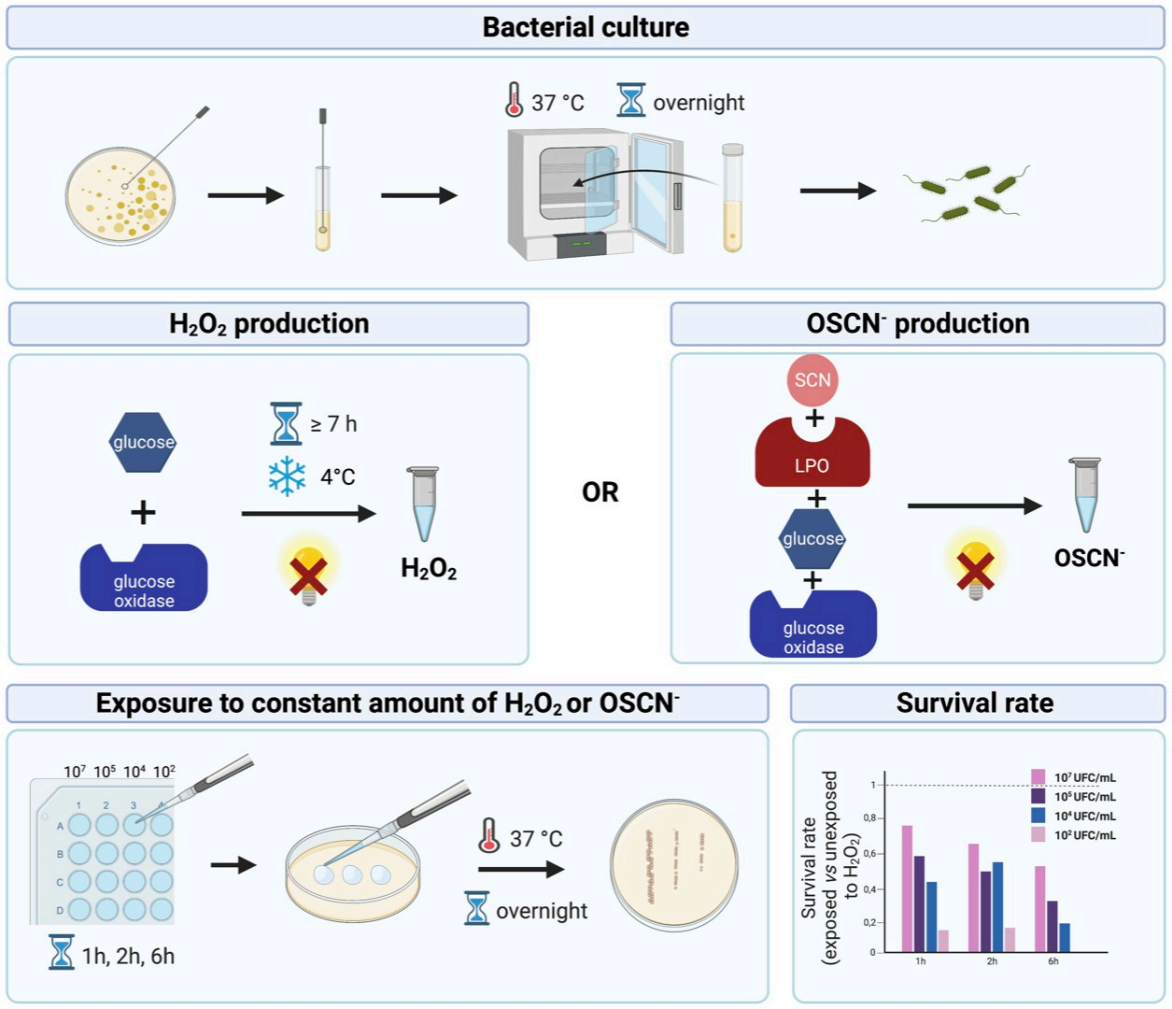
Schematic of the H_2_O_2_/LPO/SCN^-^ *in vitro* assays developed in our article. This diagram summarizes the analytic workflow for our *in vitro* test. Created in BioRender.com.

We set up and tested a model of continuous exposure to H_2_O_2_ or OSCN^-^ to improve the study of the antibacterial oxidative response in the airways (Fig 9). This response from the human airway epithelium stems from H_2_O_2_ and OSCN^-^ production.

A H_2_O_2_/LPO/SCN^-^ *in vitro* assay, which mimics the antibacterial oxidative HAE response, has been previously reported in the literature [17]. Here, we fully determined the hourly continuously produced H_2_O_2_ and OSCN^-^ concentrations within this assay. We showed that the concentrations were 500 μmol/L and 150 μmol/L for H_2_O_2_ and OSCN^-^, respectively, and stable up to 24 h. Interestingly, we found that the exposed bacteria were likely to detoxify OSCN^-^ within the H_2_O_2_/LPO/SCN^-^ *in vitro* assay, as evidenced by the decrease of OSCN^-^ concentration after 15 minutes. Therefore, we used this assay to evaluate the resistance of clinical *P*. *aeruginosa* and *S*. *aureus* strains to H_2_O_2_ and OSCN^-^. These survival rates seemed to depend on antibiotic resistance for *P*. *aeruginosa* strains but not for *S*. *aureus*.

The physiological relevance of H_2_O_2_ *in vitro* assays is somehow uncertain. First, H_2_O_2_ is used to produce OSCN^-^. Second, it has a short *in vitro* half-life of the order of a few milliseconds [4]. Although it is to date impossible to measure H_2_O_2_ directly in the human airways, several indirect methods have been used to determine its concentration. One of these methods is based on condensing expired air over 15 minutes using ice and then storing the fraction obtained in liquid nitrogen [14]. Concentrations ranging from 0 to 0.041 μmol/L were reported from healthy subjects. These concentrations might have been underestimated due to the condensation of the H_2_O_2_ fraction expired, the probable loss of some H_2_O_2_ already deteriorated by cell enzymes and its natural short half-life. The restricted period during which the air was collected can also decrease the H_2_O_2_ concentration. Another method reported in several articles relies on the measurement of production by airway epithelium cultured at the air-liquid interface (ALI). Concentrations ranging from fentomoles/20 min/mm^2^ to nanomoles/10 min/10^6^ cells were reported [15, 19]. These major *in vivo* but also *in vitro* variabilities between measures reported in the literature underline the difficulty of conceiving a relevant model. According to these data, our model might slightly overestimate the H_2_O_2_ production (500 μmol/L). It remains, however, closer to physiological ranges than a one H_2_O_2_ shot assay with higher concentrations. A caveat that is never mentioned in using such *in vitro* assay is the impact of the short H_2_O_2_ half-life, and the potential decreased concentration owing to bacterial detoxification upon exposure. Therefore, the H_2_O_2_ concentration reached in our *in vitro* H_2_O_2_ production assay might be closer to physiological levels.

As mentioned above, the OSCN^-^ production assay relies on H_2_O_2_ but also LPO and SCN^-^. For these latter, the concentrations used in the cell-free *in vitro* assay were based on those estimated physiologically [6]. It is worthy of notice that conversely to Patel *et al*. [17], we provided the hourly OSCN^-^ concentrations (~ 150 μM) obtained in our cell-free *in vitro* assay. However, this concentration has been measured in a bacteria-free environment. So, we showed that upon exposure to PA14, the OSCN^-^ concentration decreased by 85% after 15 minutes and was then stable for 1h. The short time needed for OSCN^-^ detoxification by bacteria led us to hypothesize that an enzymatic pathway was likely involved. This kind of mechanism is indeed commonly used by *Streptococcus pneumoniae* and some of the enzymes are common to both these bacteria, such as SodA [20]. But the lack of correlation between the putative enzymatic activity and the cell number in the inoculum remained unresolved. We hypothesized that H_2_O_2_ concentration decreases similarly to OSCN^-^, since H_2_O_2_ is the limiting factor for OSCN^-^ production by LPO [21]. However, we could not measure H_2_O_2_ due to bacterial interference with the ROS-Glo^™^ H_2_O_2_ Assay. This led us to propose that the optimum exposure duration was 1h, as no more ROS influenced the survival rate.

We validated the H_2_O_2_/LPO/OSCN^-^ *in vitro* assay as a bacterial inactivation test for 2 clinically relevant pathogens in pulmonary infections. We collected several clinical strains with different antibiotic resistance phenotypes. We used relevant pathophysiological bacterial inocula based on the clinical thresholds of sputum culture (10^7^ CFU/mL), bronchoalveolar lavage (10^4^ CFU/mL) and distal-protected aspirate (10^5^ CFU/mL) [22]. This latter inoculum was also tested as it is broadly used in articles, enabling our results to be compared to previous findings. All the tested inocula showed a decrease at the different hpe. However, the higher the inocula, the easier was to evidence differences at these different hpe. Therefore, we suggest using a 10^5^ CFU/mL inocula for further exploration of oxidative resistance.

**Fig 10:**
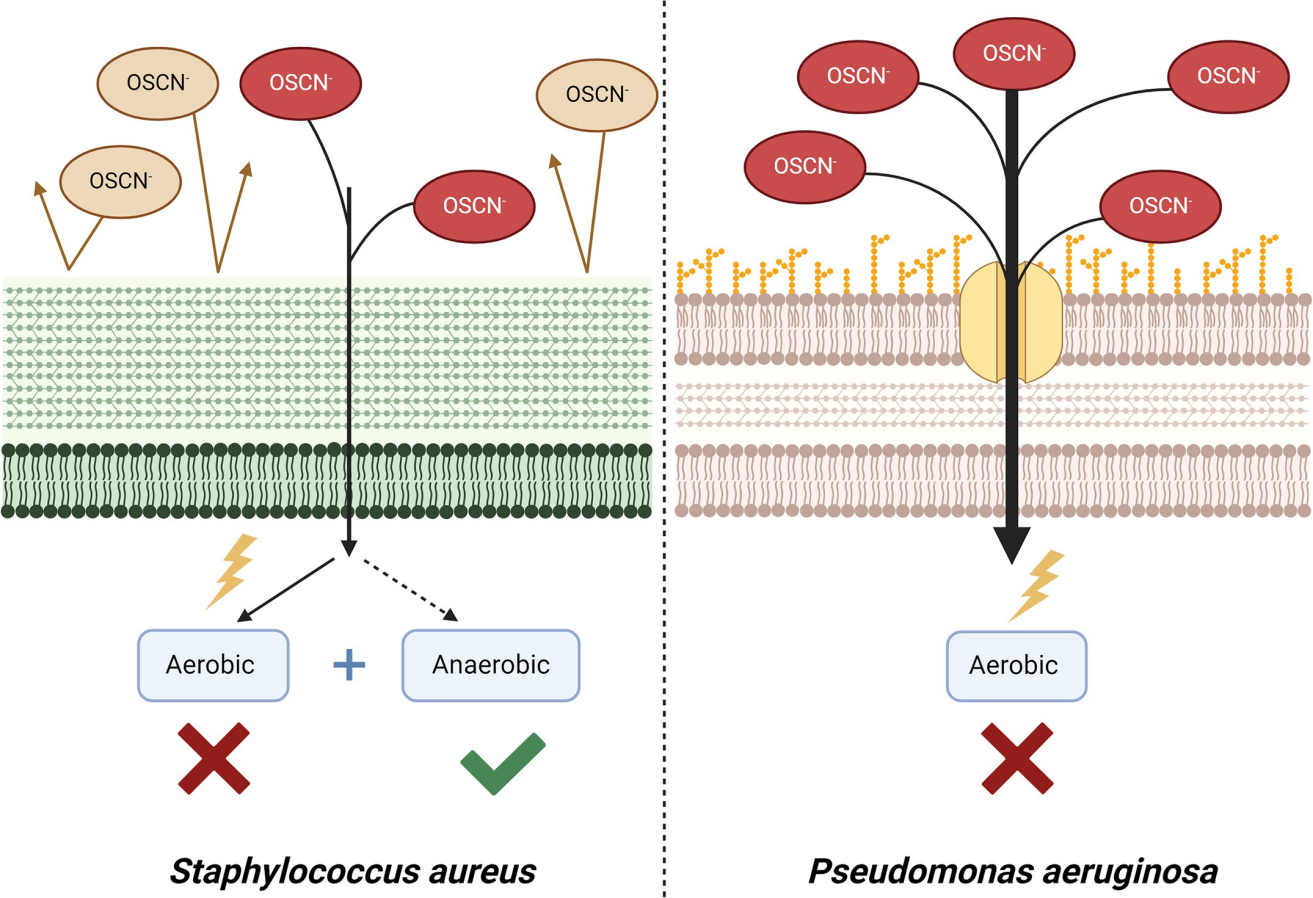
ROS action on *P*. *aeruginosa* and *S*. *aureus*. Schematic for the potential mechanisms explaining the higher sensitivity to ROS of *P*. *aeruginosa vs S*. *aureus* and *P*. *aeruginosa*. Created in BioRender.com.

Although not statistically significant, we observed that the more the *P*. *aeruginosa* strains were antibiotic-resistant, the more the survival rates decreased. This might be due to the membrane alteration, porin or fitness cost [23]. Upon H_2_O_2_ or OSCN^-^ exposure, *S*. *aureus* showed better survival rates than *P*. *aeruginosa*. This might be due to the pathway used by ROS to enter into the bacteria. Indeed, OSCN^-^ passes through porins and hydrophobic channels, these pathways being abundant on the *P*. *aeruginosa* membrane [24] (Fig 10). Moreover, given that OSCN^-^ inhibits bacterial respiration, *P*. *aeruginosa’s* susceptibility may be explained by its aerobic respiratory metabolism [25]. We found that H_2_O_2_ showed higher inactivation activity than OSCN^-^ on *P*. *aeruginosa*. We hypothesized that it could be first due to the 150 μmol/L OSCN^-^ concentration that damaged less the bacteria than the 500 μmol/L of H_2_O_2_. The OSCN^-^ concentration produced by the airway epithelium should be closer to that we obtained *in vitro* since it relies on the LPO and SCN limiting factors, which were at physiological concentrations within our model. Second, OSCN^-^ and H_2_O_2_ have very different mechanisms of action. H_2_O_2_ generates OH^-^ ions that directly affect DNA, leading to mutations that ultimately lead to cell death, while OSCN^-^ does not affect DNA but oxidizes sulfhydryl components, leading to a defect in nutrient transport [24]. This production, added to other shared oxidative mechanisms, might explain the lower survival rates obtained. These findings are key points to consider *in vitro* H_2_O_2_ or OSCN^-^ assays for assessing resistance to antibacterial oxidative response for respiratory pathogens.

## Conclusion

We elaborated an *in vitro* assay to expose bacteria to the antibacterial oxidative response of the HAE, with either H_2_O_2_ or OSCN^-^ produced continuously at physiological levels. Taking into account the concentrations produced, as well as the bacterial detoxification upon exposure, we proposed to assess optimal and relevant read-outs after 30 min or 1 hour.

Clinical antibiotic-resistant strains of *P*. *aeruginosa* seem less prompt to counteract the antibacterial oxidative response than *S*. *aureus* strains, which showed a similar ability to fight against such a response regardless of the antibiotic resistance phenotype. Further studies are needed to determine the mechanisms involved in the higher susceptibility of *P*. *aeruginosa* which is a persistent pathogen in cystic fibrosis and chronic obstructive pulmonary disease patients.

## Supporting information

S1 Fig Schematics of H_2_O_2_ (A) and OSCN^-^ (B) production assays. Created in BioRender.com.
(TIF)

S2 Fig Schematic of the synthesis of DNTB. Production of TNB and measurement of OSCN^-^ concentration (DTNB is cleaved thanks to sodium borohydride (NaBH_4_), TNB then combines with OSCN^-^ to form a TNB-OSCN).
(TIF)

S3 Fig HR-ESI-MS spectrum of TNB (A) and HR-ESI-MS spectrum of DTNB (B). (TIF)

S1 Data Raw data of experiments performed in this study. (XLSX)

S1 File Statistical analysis for figures. (DOCX)
