# Supplementary figures and images for: Resistance of Pseudomonas aeruginosa and Staphylococcus aureus to the airway epithelium oxidative response assessed by a cell-free in vitro assay
Source: PLoS One. 2024 Aug 14;19(8):e0306259. doi: 10.1371/journal.pone.0306259 (PMC11324103; doi:10.1371/journal.pone.0306259)

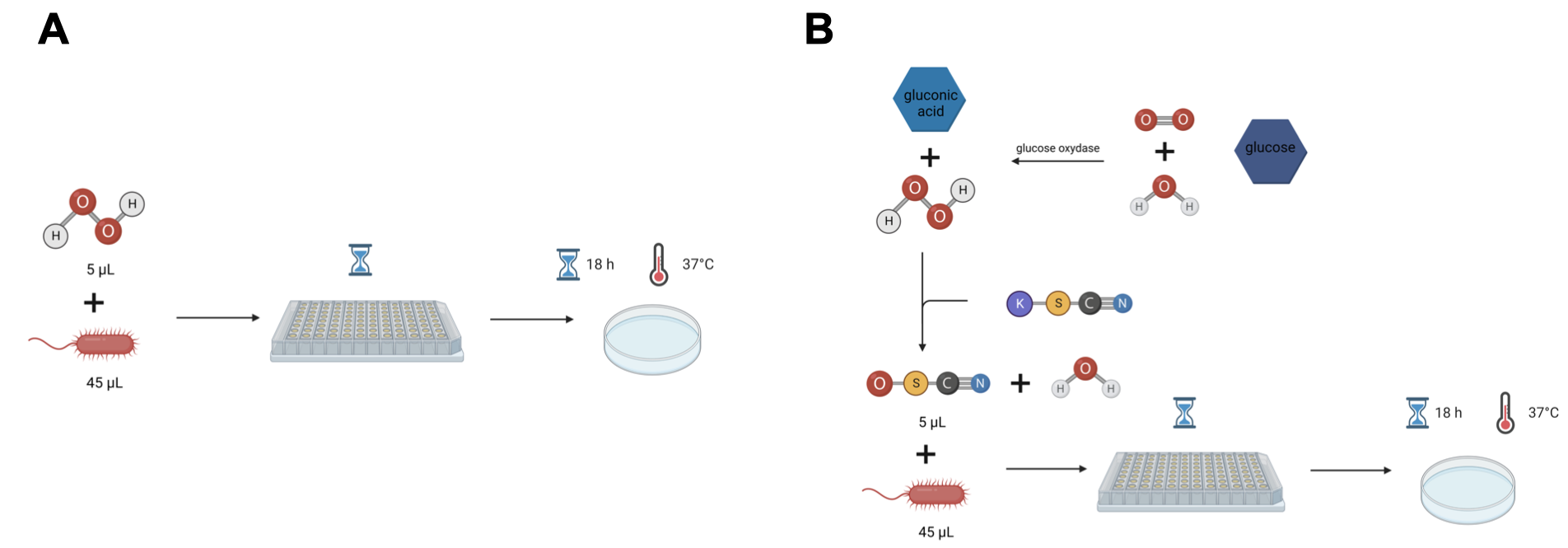

Supplement: S1 Fig — Created in BioRender.com. (TIF) [file pone.0306259.s001.tif]

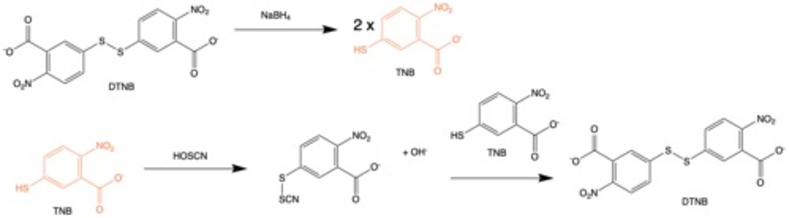

Supplement: S2 Fig — Production of TNB and measurement of OSCN- concentration (DTNB is cleaved thanks to sodium borohydride (NaBH4), TNB then combines with OSCN- to form a TNB-OSCN). (TIF) [file pone.0306259.s002.tif]

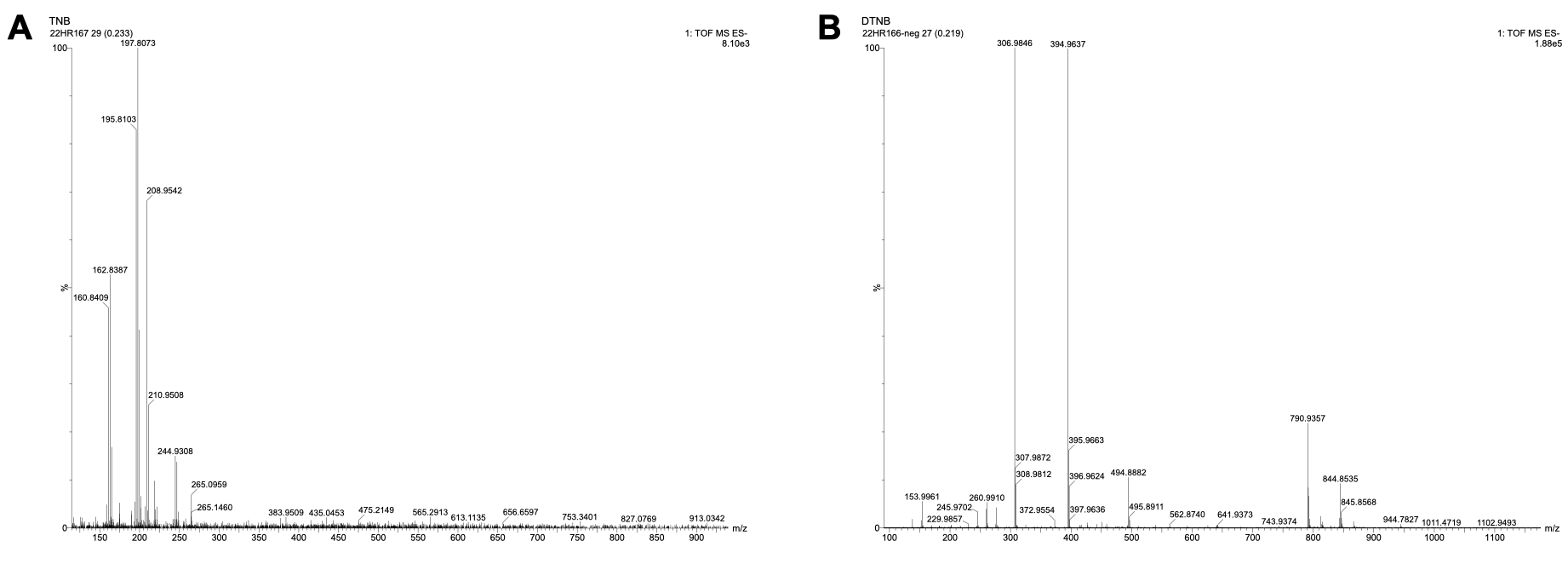

Supplement: S3 Fig — (TIF) [file pone.0306259.s003.tif]
