# Supplementary material for: Resistance of Pseudomonas aeruginosa and Staphylococcus aureus to the airway epithelium oxidative response assessed by a cell-free in vitro assay
Source: PLoS One. 2024 Aug 14;19(8):e0306259. doi: 10.1371/journal.pone.0306259 (PMC11324103; doi:10.1371/journal.pone.0306259)
Supplement: S1 File — (DOCX) [file pone.0306259.s005.docx]

**Supporting information**

**Resistance of *Pseudomonas aeruginosa* and *Staphylococcus aureus* to the airway epithelium oxidative response assessed by a cell-free *in vitro* assay**

Maïwenn Petithomme-Nanrocki^1^, Nathan Nicolau-Guillaumet^2^, Nicolas Borie^3^, Arnaud Haudrechy^3^, Jean-Hugues Renault^3^, Sophie Moussalih^1^, Anaëlle Muggeo^2^, Thomas Guillard^2,*^

^1^ Université de Reims Champagne-Ardenne, INSERM, P3Cell, U 1250, Reims, France

^2^ Université de Reims Champagne-Ardenne, INSERM, CHU de Reims, Laboratoire de bactériologie-Virologie-Hygiène hospitalière-Parasitologie-Mycologie, P3Cell, U 1250, Reims, France

^3^ Université de Reims Champagne-Ardenne, CNRS, ICMR, UMR 7312, Reims, France.

# Statistical analysis for Figures

**Figure 4. Survival rates of PA14 within the H_2_O_2_/LPO/SCN- *in vitro* assays.**

For H_2_O_2_, the mean difference for 10^4^ CFU/mL at 4 hpe and 6 hpe compared to 1 hpe were 0.2418 and 0.1697, respectively; 95% CI of difference [0.08559; 0.398] and [0.01347; 0.3259]. For OSCN-, the mean difference for 104 CFU/mL at 6 hpe compared to 1 hpe was 0.2573; 95% CI of difference [0.05879; 0.4557].

**Figure 5. The survival rate of reference and susceptible clinical *P. aeruginosa* strains within the H_2_O_2_ and the H_2_O_2_/LPO/SCN^-^ *in vitro* assays.**

For ATCC 27853 exposed to H_2_O_2_, the mean difference for 10^4^ and 10^5^ CFU/mL at 6 hpe compared to 1 hpe were 0.1913 and 0.177 respectively; 95% CI of difference [0.04931; 0.3332] and [0.03498; 0.3189]. For ATCC 27853 exposed to OSCN^-^, the mean difference for 10^2^, 10^4^, 10^5^ and 10^7^ CFU/mL at 6 hpe compared to 1 hpe were 0.3, 0.2152, 0.1782 and 0.1898 respectively; 95% CI of difference [0.1789; 0.4211], [0.1003; 0.3301], [0.06328; 0.293] and [0.07496; 0.3047]. For RSR 2020609 exposed to H_2_O_2_, the mean difference for 10^2^, 10^4^ and 10^5^ CFU/mL at 6 hpe compared to 1 hpe were 0.2083, 0.1952 and 0.1854 respectively; 95% CI of difference [0.06672; 0.3499], [0.05361; 0.3368] and [0.04383; 0.3271]. For RSR 2020609 exposed to OSCN^-^, the mean difference for 10^2^, 10^4^, 10^5^ and 10^7^ CFU/mL at 6 hpe, compared to 1 hpe were 02896, 0.2874, 0.2566 and 0.2352, respectively; 95% CI of difference [0.1611; 0.4182], [0.1589; 0.4159], [0.1281; 0.3852] and [0.1067; 0.3638]; for 10^2^ and 10^4^ CFU/mL at 2 hpe compared to 1 hpe were 0.1814 and 0.1298 respectively; 95% CI of difference [0.05289; 0.31] and [0.00122; 0.2583].

**Figure 6. Survival rate of resistant clinical *P. aeruginosa* strains within the H_2_O_2_ and the H_2_O_2_/LPO/SCN^-^ *in vitro* assays.**

For RSR 2020610 exposed to H_2_O_2_, the mean difference for 10^2^ at 2 hpe and 6 hpe, and for 10^5^ CFU/mL at 6 hpe compared to 1 hpe were 0.3111, 0.2917 and 0.1617 respectively; 95% CI of difference [0.1572; 0.465], [0.1378; 0.4455] and [0.007798; 0.3155]. For RSR 2020610 exposed to OSCN^-^, the mean difference for 10^2^, 10^4^, 10^5^ and 10^7^ CFU/mL at 6 hpe compared to 1 hpe were 0.3115, 0.2747, 0.2995 and 0.2796 respectively; 95% CI of difference [0.2046; 0.4185], [0.1003; 0.3301], [0.1678; 0.3817], [0.1926 to 0.4065] and [0.1726 to 0.3865]; for 10^2^, 10^4^ and 10^7^ CFU/mL at 2 hpe compared to 1 hpe were 0.2174, 0.1208 and 0.1292 respectively; 95% CI of difference [0.1105; 0.3244], [0.01386; 0.2278] and [0.02227; 0.2362]. For RSR 2020611 exposed to H_2_O_2_, the mean difference for 10^4^, 10^5^ and 10^7^ CFU/mL at 6 hpe compared to 1 hpe were 0.2601, 0.2735, and 0.1765 respectively; 95% CI of difference [0.1071; 0.413], [0.1206; 0.4265] and [0.02353; 0.3295]; for 10^5^ CFU/mL at 2 hpe compared to 1 hpe were 0.1981; 95% CI of difference [0.04513; 0.3511]. For RSR 2020611 exposed to OSCN, the mean difference for 10^2^, 10^4^, 10^5^ and 10^7^ CFU/mL at 6 hpe compared to 1 hpe were 0.1876, 0.1981, 0.1961 and 0.1943 respectively; 95% CI of difference [0.1012; 0.274], [0.1117; 0.2845], [0.1097; 0.2825] and [0.1079; 0.2807]; for 10^2^, 10^4^ and 10^7^ CFU/mL at 2 hpe compared to 1 hpe were 0.1876, 0.1172 and 0.107; 95% CI of difference [0.1012; 0.274], [0.03085; 0.2036] and [0.02057; 0.1934]. For RSR 2020612 exposed to H_2_O_2_, the mean difference for 10^4^, 10^5^ and 10^7^ CFU/mL at 6 hpe compared to 1 hpe were 0.2517, 0.2615, and 0.1708 respectively; 95% CI of difference [0.1162; 0.3873], [0.126; 0.3971] and [0.03523; 0.3063]; for 10^4^ and 10^5^ CFU/mL at 2 hpe compared to 1 hpe were 0.1706 and 0.1499; 95% CI of difference [0.03509; 0.3062] and [0.01435; 0.2854]. For RSR 2020612 exposed to OSCN^-^, the mean difference for 10^4^, 10^5^ and 10^7^ CFU/mL at 6 hpe compared to 1 hpe were 0.2372, 0.2235 and 0.2218 respectively; 95% CI of difference [0.1694; 0.305], [0.1557; 0.2914] and [0.154; 0.2897]; for 10^4^, 10^5^ and 10^7^ CFU/mL at 2 hpe compared to 1 hpe were 0.1076, 0.1031 and 0.1107; 95% CI of difference [0.03979; 0.1754], [0.0353; 0.1709] and [0.04283; 0.1785]. For RSR 2020911 exposed to H_2_O_2_, the mean difference for 10^4^, 10^5^ and 10^7^ CFU/mL at 6 hpe compared to 1 hpe were 0.2108, 0.2526 and 0.1923 respectively; 95% CI of difference [0.08432; 0.3374], [0.126; 0.3791] and [0.06576; 0.3188]; for 10^4^, 10^5^ and 10^7^ CFU/mL at 2 hpe compared to 1 hpe were 0.1417, 0.2022 and 0.1555; 95% CI of difference [0.01515; 0.2682], [0.07566; 0.3287] and [0.02901; 0.2821]. For RSR 2020911 exposed to OSCN^-^, the mean difference for 10^2^, 10^4^, 10^5^ and 10^7^ CFU/mL at 6 hpe compared to 1 hpe were 0.2014, 0.3125, 0.2861 and 0.2423 respectively; 95% CI of difference [0.09529; 0.3076], [0.2063; 0.4186], [0.1799; 0.3922] and [0.1361; 0.3484]; for 10^2^, 10^4^ and 10^5^ CFU/mL at 2 hpe compared to 1 hpe were 0.1529, 0.1062 and 0.1091; 95% CI of difference [0.04671; 0.259], [1.16x10^-005^; 0.2123] and [0.002991; 0.2153].

**Figure 7. Survival rates of reference and clinical methicillin-susceptible *S. aureus* strains within the H_2_O_2_ and H_2_O_2_/LPO/SCN^-^ *in vitro* assays.**

For ATCC 20213 exposed to OSCN^-^, the mean difference for 10^4^, 10^5^ and 10^7^ at 6 hpe compared to 1 hpe were 0.2359, 0.3738 and 0.336, respectively; 95% CI of difference [0.09746; 0.3744], [0.2353; 0.5123], [0.2461; 0.5863] and [0.1975; 0.4745]. For RSR 2022617 exposed to H_2_O_2_, the mean difference for 10^4^, 10^5^ and 10^7^ CFU/mL at 6 hpe compared to 1 hpe were 0.4578, 0.5261, and 0.3533 respectively; 95% CI of difference [0.2889; 0.6266], [0.3572; 0.6949] and [0.1845; 0.5222]. For RSR 2022617 exposed to H_2_O_2_, the mean difference for 10^4^, 10^5^ and 10^7^ CFU/mL at 6 hpe compared to 1 hpe were 0.4578, 0.5261, and 0.3533 respectively; 95% CI of difference [0.2889; 0.6266], [0.3572; 0.6949] and [0.1845; 0.5222]; for 10^4^ at 2 hpe compared to 1 hpe was 0.1973; 95% CI of difference [0.07683; 0.3178]. For RSR 2022616 exposed to H_2_O_2_, the mean difference for 10^2^, 10^4^, 10^5^ and 10^7^ CFU/mL at 6 hpe compared to 1 hpe were 0.17, 0.313, 0.3711 and 0.2856 respectively; 95% CI of difference [0.0378; 0.3022], [0.1808; 0.4452], [0.2389; 0.5033] and [0.1534; 0.4178]. For RSR 2022616 exposed to OSCN^-^, the mean difference for 10^2^, 10^4^, 10^5^ and 10^7^ CFU/mL at 6 hpe compared to 1 hpe were 0.2676, 0.3974, 0.3194 and 0.2824 respectively; 95% CI of difference [0.1252; 0.4095], [0.2553; 0.5395], [0.1772; 0.4615] and [0.1402; 0.4245]; for 10^2^ CFU/mL at 2 hpe compared to 1 hpe were 0.2143; 95% CI of difference [0.0722; 0.3565]. For RSR 2022618 exposed to H_2_O_2_, the mean difference for 10^4^, 10^5^ and 10^7^ CFU/mL at 6 hpe compared to 1 hpe were 0.3868, 0.4555, and 0.3465 respectively; 95% CI of difference [0.2539; 0.5197], [0.3226; 0.5884] and [0.2136; 0.4794]; for 10^5^ CFU/mL at 2 hpe compared to 1 hpe were 0.1935; 95% CI of difference [0.06059; 0.3264]. For RSR 2022618 exposed to OSCN^-^, the mean difference for 10^4^, 10^5^ and 10^7^ CFU/mL at 6 hpe compared to 1 hpe were 0.2767, 0.2591 and 0.3377 respectively; 95% CI of difference [0.1739; 0.3795], [0.1563; 0.3619] and [0.2349; 0.4405].

**Figure 8. Survival rates of reference and clinical methicillin-resistant *S. aureus* strains within the H_2_O_2_ and H_2_O_2_/LPO/SCN^-^ *in vitro* assays.** For NCTC12493 exposed to H_2_O_2_, the mean difference for 10^2^, 10^4^, 10^5^ and 10^7^ at 6 hpe compared to 1 hpe were 0.2, 0.3956, 0.513 and 0.5038 respectively; 95% CI of difference [0.03637; 0.3636], [0.2319; 0.5592], [0.3493; 0.6766] and [0.3402; 0.6675]; for 10^2^, 10^4^ and 10^5^ at 2 hpe compared to 1 hpe were 0.2, 0.1645 and 0.2108 respectively; 95% CI of difference [0.03637; 0.3636], [0.000821; 0.3281] and [0.04714; 0.3744]. For NCTC12493 exposed to OSCN^-^, the mean difference for 10^4^, 10^5^ and 10^7^ at 6 hpe compared to 1 hpe were 0.6593, 0.5014 and 0.5707, respectively; 95% CI of difference [0.5256; 0.793], [0.3677; 0.6351] and [0.4371; to 0.7044]; for 10^4^, 10^5^ and 10^7^ at 2 hpe compared to 1 hpe were 0.2396, 0.171 and 0.14 respectively; 95% CI of difference [0.1059; 0.3733], [0.03731; 0.3047] and [0.006303; 0.2737]. For RSR 20220620 exposed to H_2_O_2_, the mean difference for 10^2^, 10^4^, 10^5^ and 10^7^ CFU/mL at 6 hpe compared to 1 hpe were 0.1333, 0.3288, 0.3587 and 0.462 respectively; 95% CI of difference [0.01046; 0.2562], [0.2059; 0.4517], [0.2358; 0.4816] and [0.3392; 0.5849]; for 10^5^ and 10^7^ CFU/mL at 2 hpe compared to 1 hpe were 0.1375 and 0.1613 respectively; 95% CI of difference [0.01466; 0.2604] and [0.03841; 0.2842]. For RSR 20220620 exposed to OSCN^-^, the mean difference for 10^4^, 10^5^ and 10^7^ CFU/mL at 6 hpe compared to 1 hpe were 0.3842, 0.3443 and 0.3046 respectively; 95% CI of difference [0.2452; 0.5232], [0.2053; 0.4833] and [0.1656; 0.4436]. For RSR 20220619 exposed to H_2_O_2_, the mean difference for 10^4^, 10^5^ and 10^7^ CFU/mL at 6 hpe compared to 1 hpe were 0.3682, 0.3841 and 0.3645 respectively; 95% CI of difference [0.2605; 0.4759], [0.2764; 0.4918] and [0.2568; 0.4722]; for 10^4^ and 10^5^ CFU/mL at 2 hpe compared to 1 hpe were 0.1426 and 0.1351 respectively; 95% CI of difference [0.03495; 0.2503] and [0.02747; 0.2428]. For RSR 20220619 exposed to OSCN^-^, the mean difference for 10^2^, 10^4^, 10^5^ and 10^7^ CFU/mL at 6 hpe compared to 1 hpe were 0.2188, 0.1938, 0.2058 and 0.1479 respectively; 95% CI of difference [0.09609; 0.3416], [0.2553; 0.5395], [0.07102; 0.3165], [0.08308; 0.3286] and [0.02516; 0.2706]; for 10^2^ CFU/mL at 2 hpe compared to 1 hpe were 0.2045; 95% CI of difference [0.0818; 0.3273].
